# The Impact of Oxidative Stress on Male Reproductive Function: Exploring the Role of Antioxidant Supplementation

**DOI:** 10.7759/cureus.42583

**Published:** 2023-07-27

**Authors:** Gireeja Walke, Sagar S Gaurkar, Roshan Prasad, Tejaswee Lohakare, Mayur Wanjari

**Affiliations:** 1 Department of Medicine, Jawaharlal Nehru Medical College, Datta Meghe Institute of Higher Education and Research, Wardha, IND; 2 Department of Otolaryngology - Head and Neck Surgery, Jawaharlal Nehru Medical College, Datta Meghe Institute of Higher Education and Research, Wardha, IND; 3 Department of Internal Medicine, Jawaharlal Nehru Medical College, Datta Meghe Institute of Higher Education and Research, Wardha, IND; 4 Department of Child Health Nursing, Smt. Radhikabai Meghe Memorial College of Nursing, Datta Meghe Institute of Higher Education and Research, Wardha, IND; 5 Department of Research and Development, Jawaharlal Nehru Medical College, Datta Meghe Institute of Higher Education and Research, Wardha, IND

**Keywords:** infertility, dna damage, fertility, sperm quality, antioxidant supplementation, male reproductive function, oxidative stress

## Abstract

Male reproductive function is highly susceptible to oxidative stress, which arises from an imbalance between reactive oxygen species (ROS) production and antioxidant defense mechanisms. Oxidative stress can significantly impair sperm quality, including count, motility, morphology, and DNA integrity, leading to male infertility. Antioxidants play a crucial role in maintaining reproductive health by neutralizing ROS and protecting sperm cells from oxidative damage. This review article explores the impact of oxidative stress on male reproductive function and investigates the potential benefits of antioxidant supplementation in mitigating its detrimental effects. A comprehensive literature search was conducted to gather relevant studies examining the effects of oxidative stress on male fertility and the outcomes of antioxidant supplementation. The findings reveal that antioxidant supplementation can improve sperm quality, DNA integrity, and fertility outcomes in some individuals. However, conflicting research findings and limitations in study design highlight the need for further investigation. Factors such as individual variations, underlying causes of infertility, dosage, and duration of supplementation should be carefully considered. Lifestyle modifications, including a healthy diet and exercise, are crucial in reducing oxidative stress and optimizing male reproductive health. This review article provides valuable insights into the complex relationship between oxidative stress and male reproductive function, emphasizing the potential role of antioxidant supplementation as a supportive strategy. Further research is warranted to establish optimal protocols, identify specific subgroups that may benefit the most, and explore advancements in antioxidant therapies to improve male fertility outcomes.

## Introduction and background

Oxidative stress occurs when there is an imbalance between the production of reactive oxygen species (ROS) and the body's ability to neutralize or repair their damaging effects. While ROS are essential for various physiological processes, excessive levels can lead to oxidative damage and disrupt cellular homeostasis. Oxidative stress has been implicated in numerous health conditions, including male reproductive dysfunction [1]. The male reproductive system is highly susceptible to oxidative stress due to its unique composition and physiological processes. Spermatozoa are particularly vulnerable to oxidative damage as they possess limited antioxidant defense mechanisms. The delicate balance between ROS production and antioxidant protection is crucial for maintaining optimal sperm function and fertility [2,3].

Understanding the impact of oxidative stress on male reproductive function is paramount due to the rising incidence of male infertility worldwide. Infertility affects approximately 10% to 15% of couples globally, with male factors contributing to nearly 50% of these cases. Oxidative stress is a potential underlying mechanism contributing to male infertility [4]. Investigating the role of oxidative stress in male reproductive function can provide valuable insights into the etiology and management of male infertility. It can help identify novel therapeutic targets and interventions to preserve and improve sperm quality, ultimately enhancing fertility outcomes [5].

This review article aims to comprehensively explore the impact of oxidative stress on male reproductive function, with a particular focus on the role of antioxidant supplementation. By examining the current body of scientific evidence, we aim to shed light on the mechanisms through which oxidative stress affects sperm quality and fertility. Additionally, this article seeks to investigate the potential benefits of antioxidant supplementation in mitigating the adverse effects of oxidative stress on male reproductive health. We will delve into the various types of antioxidants commonly used in supplementation, their proposed mechanisms of action, and the scientific evidence supporting their use. By synthesizing existing research findings, this article intends to provide a comprehensive understanding of the interplay between oxidative stress and male reproductive function. Furthermore, it aims to offer practical insights and recommendations for clinicians and researchers interested in optimizing male fertility through antioxidant interventions.

## Review

Methodology

A comprehensive review of the impact of oxidative stress on male reproductive function and the role of antioxidant supplementation was conducted using a systematic literature search. Relevant articles were identified by searching electronic databases such as PubMed, Scopus, and Web of Science using keywords and combinations related to oxidative stress, male reproductive function, sperm quality, and antioxidant supplementation. No language or time restrictions were applied, and original research articles and review papers were considered. Additionally, reference lists of retrieved articles were examined to identify any additional relevant studies. The inclusion criteria involved selecting studies relevant to the topic, including human subjects (fertile and infertile males), employing appropriate study designs, and focusing on outcome measures related to sperm parameters, DNA damage, and fertility outcomes. Only peer-reviewed articles published in reputable journals were included to ensure quality and validity. Studies solely focusing on female reproductive health or unrelated aspects of oxidative stress were excluded. The screening process initially assessed titles and abstracts, followed by a thorough evaluation of full-text articles based on the inclusion and exclusion criteria. Any uncertainties or discrepancies in study selection were resolved through reviewer discussions. By applying these rigorous criteria, the selected studies incorporated in this review article are reliable and pertinent to the topic at hand.

Understanding oxidative stress

Definition and Causes of Oxidative Stress

Oxidative stress is a physiological condition characterized by excessive production of ROS and a diminished ability of the body's antioxidant defense system to neutralize them effectively. ROS are highly reactive molecules, including free radicals like superoxide anion (O2·), hydroxyl radical (OH·), and nonradical species like hydrogen peroxide (H_2_O_2_). They are natural byproducts of cellular metabolism and play important roles in cell signaling, immune responses, and other physiological processes [6]. However, when ROS production exceeds the body's capacity to neutralize them, it can lead to oxidative damage of lipids, proteins, and DNA. This damage can disrupt cellular functions and contribute to various diseases, including male infertility [7].

The main sources of ROS production in the male reproductive system are discussed next.

Spermatozoa: Sperm cells themselves are a significant source of ROS production. During normal sperm physiology, ROS generation occurs as part of the process of capacitation and hyperactivation, which are essential for sperm function and fertilization. However, excessive ROS production by sperm cells can lead to oxidative damage to their membranes, DNA, and other cellular components, resulting in impaired sperm motility, reduced viability, and compromised fertilization capacity [8].

Leukocytes: Immune cells, such as leukocytes, are present in the male reproductive tract and can produce ROS as a defense mechanism against infections. Inflammatory processes and infections can stimulate leukocytes to generate ROS to combat pathogens. However, under certain conditions, such as chronic infections or inflammation, the production of ROS by leukocytes can become excessive and lead to oxidative stress. This can negatively impact sperm function and contribute to male infertility [9].

Seminal plasma: Seminal plasma, which surrounds and supports sperm cells, contains various enzymes and molecules that can generate ROS. These include enzymes such as nicotinamide adenine dinucleotide phosphate (NADPH) oxidase and xanthine oxidase and molecules like prostaglandins and lipid peroxides. The presence of these components in seminal plasma contributes to the overall oxidative environment in the male reproductive tract. While ROS production in seminal plasma serves physiological functions, excessive ROS levels can overwhelm antioxidant defenses and induce oxidative stress, affecting sperm quality and fertility [10]. Understanding the sources of ROS production in the male reproductive system is crucial for comprehending the mechanisms underlying oxidative stress and its impact on male fertility. Targeting these sources and maintaining a balance between ROS production and antioxidant defense mechanisms may mitigate the negative effects of oxidative stress on male reproductive function and improve fertility outcomes.

Mechanisms of Oxidative Stress in the Male Reproductive System

The male reproductive system is particularly susceptible to oxidative stress, primarily attributed to the unique characteristics of sperm cells. Sperm cell membranes contain highly unsaturated fatty acids, making them highly vulnerable to ROS attack. Additionally, spermatozoa have limited antioxidant enzyme systems, further exacerbating their susceptibility to oxidative damage [11].

Lipid peroxidation is one of the key mechanisms through which oxidative stress affects male reproductive function. ROS attacks the polyunsaturated fatty acids in sperm cell membranes, initiating a chain reaction known as lipid peroxidation. This process leads to the production of lipid peroxides and the disruption of membrane integrity. As a result, the functionality and viability of sperm are compromised, leading to impaired motility and reduced fertilization capacity [12].

Another mechanism by which oxidative stress impacts male reproductive function is through protein oxidation. ROS can oxidatively modify proteins in sperm, leading to structural alterations and functional impairments. This oxidative damage to proteins can affect various aspects of sperm physiology, including motility, DNA packaging, and fertilization ability [13].

Furthermore, oxidative stress can induce DNA damage in sperm cells. Sperm DNA is highly compacted and tightly packaged within the nucleus, making it more susceptible to oxidative damage. ROS can directly attack and cause oxidative lesions in the DNA strand, leading to DNA fragmentation and impaired genetic integrity. Such DNA damage in sperm has been associated with reduced fertilization rates, decreased embryo quality, and an increased risk of developmental abnormalities in offspring [14]. These mechanisms collectively highlight oxidative stress's significant impact on male reproductive function. The lipid peroxidation of sperm cell membranes, protein oxidation, and DNA damage contributes to impaired sperm motility, reduced fertility potential, and an increased risk of infertility. Understanding these mechanisms is crucial in elucidating the role of oxidative stress in male infertility. It emphasizes the need for strategies to mitigate oxidative damage, such as antioxidant supplementation, to preserve and enhance male reproductive health [15].

Effects of Oxidative Stress on Sperm Quality and Fertility

The adverse effects of oxidative stress on sperm quality and fertility are well-documented, highlighting the importance of understanding its mechanisms and potential interventions. Elevated levels of ROS can induce various detrimental effects on sperm, profoundly impacting male reproductive function. These effects include reduced sperm motility, where oxidative stress impairs the flagellar movement, leading to decreased forward progression and diminished capacity to reach and penetrate the egg during fertilization [16].

Furthermore, oxidative stress can cause structural abnormalities in sperm, resulting in abnormal sperm morphology and negatively affecting their fertilization potential. Additionally, increased oxidative stress can lead to a decline in sperm viability, as it induces sperm cell death through apoptosis, ultimately reducing the overall sperm count and fertility [17].

One of the most concerning consequences of oxidative stress in sperm is the induction of DNA damage. ROS-induced DNA fragmentation in sperm is associated with reduced fertilization rates and an increased risk of miscarriage, posing significant challenges to successful reproduction. Moreover, oxidative stress can impair fertilization rates and embryonic development, ultimately reducing pregnancy success and affecting the establishment of a healthy pregnancy [18]. Understanding these oxidative stress mechanisms on male reproductive function is vital for identifying potential interventions to preserve and enhance sperm quality. In the subsequent section, we will delve into the role of antioxidants in reproductive health and explore how they may act as protective agents, counteracting the harmful effects of oxidative stress on male fertility. By gaining insights into the interplay between oxidative stress and antioxidants, we can explore new avenues for improving male reproductive health and addressing infertility issues [19].

Role of antioxidants in reproductive health

Definition and Types of Antioxidants

Antioxidants are compounds that can neutralize or inhibit the harmful effects of ROS, thereby reducing oxidative stress. They work by either directly scavenging free radicals or by enhancing the activity of the body's endogenous antioxidant defense system. Various types of antioxidants play important roles in maintaining reproductive health.

Enzymatic antioxidants: Enzymatic antioxidants play a crucial role in the defense against oxidative stress. They include enzymes such as superoxide dismutase (SOD), catalase, and glutathione peroxidase (GPx). SOD catalyzes the conversion of superoxide radicals into H_2_O_2_, while catalase and GPx further break down H_2_O_2_ into water and oxygen. By facilitating these enzymatic reactions, they effectively neutralize ROS and prevent their harmful effects on sperm cells [20].

Nonenzymatic antioxidants: Nonenzymatic antioxidants comprise a wide range of compounds crucial in directly scavenging free radicals and safeguarding against oxidative damage. This diverse category includes vitamins, minerals, carotenoids, and flavonoids. Notably, certain vitamins, such as vitamins C and E, exhibit potent antioxidant properties by donating electrons to neutralize free radicals. In addition to vitamins, minerals like selenium and zinc are equally indispensable in maintaining the antioxidant defense system and supporting the activity of enzymatic antioxidants. These minerals act as coenzymes essential for various metabolic enzymatic reactions, contributing significantly to the overall antioxidant defense mechanism. Carotenoids, like beta-carotene and lycopene, also play a vital role in this category by possessing robust antioxidant properties that effectively shield against lipid peroxidation. Furthermore, flavonoids, found abundantly in fruits and vegetables, demonstrate antioxidant effects and reduce oxidative stress [21].

By integrating the actions of both enzymatic and nonenzymatic antioxidants, the body establishes a powerful defense system against oxidative stress. Through a synergistic effect, these antioxidants work harmoniously to scavenge free radicals, prevent lipid peroxidation, and protect sperm cells from oxidative damage. Maintaining an adequate intake of these antioxidants through a balanced diet or supplementation can play a pivotal role in sustaining the redox balance, promoting optimal sperm quality, and supporting overall male reproductive health.

Importance of Antioxidants in Maintaining Reproductive Function

Protection of sperm integrity: One of the key benefits of antioxidant supplementation is the protection of sperm cell integrity. Antioxidants help inhibit lipid peroxidation, which damages the sperm cell membranes. By preventing lipid peroxidation, antioxidants maintain the structural integrity of the sperm cells, ensuring optimal sperm motility, viability, and fertilization potential [22].

DNA protection: Oxidative stress can induce DNA damage in sperm, leading to DNA fragmentation and genetic abnormalities. Antioxidants play a crucial role in protecting sperm DNA from oxidative damage. By scavenging free radicals and reducing oxidative stress, antioxidants minimize the risk of DNA fragmentation, thereby preserving the genetic integrity of sperm and reducing the potential negative impact on offspring [23].

Enhancement of sperm quality: Antioxidant supplementation has improved various aspects of sperm quality. Studies have demonstrated that antioxidants can increase sperm count, improve sperm motility, enhance sperm morphology, and positively impact overall semen quality. By reducing oxidative stress, antioxidants help optimize sperm parameters, improving reproductive outcomes and increasing the chances of successful fertilization [24].

Preservation of fertility potential: Antioxidants preserve fertility potential, particularly in age-related decline. As individuals age, oxidative stress increases, decreasing sperm quality and fertility. It depends on other factors, such as thyroid-stimulating hormone (TSH) and testosterone. Antioxidant supplementation can help counteract age-related oxidative damage, maintaining the functional competence of sperm and preserving fertility. Additionally, antioxidants can mitigate the negative impact of environmental factors and lifestyle choices, such as exposure to pollutants and smoking, on male reproductive health [25].

Sources of Antioxidants in the Diet

Fruits and vegetables: Incorporating a variety of colorful fruits and vegetables into your diet is an excellent way to increase antioxidant intake. Like blueberries, strawberries, and raspberries, berries are rich in antioxidants like anthocyanins. Citrus fruits, like oranges and grapefruits, provide vitamin C, a powerful antioxidant. Tomatoes are a good source of lycopene, while leafy greens like spinach and broccoli offer vitamins C and E and other phytochemicals with antioxidant properties. Bell peppers also contain antioxidants, including vitamin C [26].

Nuts and seeds: Nuts and seeds are a great source of healthy fats and protein and contain antioxidants. Almonds and walnuts, for example, provide vitamin E, a potent antioxidant that helps protect cells from oxidative damage. Flaxseeds and chia seeds are rich in antioxidants called lignans, associated with various health benefits [27].

Whole grains: Whole grains such as brown rice, quinoa, and whole wheat are good sources of fiber and contain antioxidants. These antioxidants include selenium, which has been shown to have a protective effect against oxidative stress, and various phytochemicals that contribute to the overall antioxidant capacity of whole grains [28].

Legumes: Legumes, including beans, lentils, and chickpeas, offer a range of health benefits, including antioxidants. They contain flavonoids, a group of antioxidants associated with reduced risk of chronic diseases. Legumes are also a great source of vitamins, minerals, and fiber, making them valuable to a balanced diet [29].

Herbs and spices: Herbs and spices flavor dishes and provide antioxidant benefits. Turmeric, known for its active compound curcumin, has potent antioxidant and anti-inflammatory properties. Cinnamon, ginger, garlic, and oregano are also rich in antioxidants and can be used to enhance the antioxidant content of meals [30]. While a balanced diet should be the primary source of antioxidants, antioxidant supplementation may be considered in some cases. In the next section, we will explore the impact of oxidative stress on male reproductive function in more detail, focusing on sperm count, motility, morphology, and DNA integrity.

The impact of oxidative stress on male reproductive function

Effects of Oxidative Stress on Sperm Count, Motility, and Morphology

Sperm count: Oxidative stress has been linked to decreased sperm count, a condition known as oligospermia. High levels of ROS can induce oxidative damage to sperm cells, leading to cell death through apoptosis. This cell death can reduce overall sperm count, affecting male fertility [31].

Sperm motility: Oxidative stress can impair sperm motility, which refers to the ability of sperm to move and swim effectively. ROS can affect the flagellar movement of sperm, reducing their forward progression and hindering their ability to navigate through the female reproductive tract. The reduction in sperm motility, known as asthenospermia, can significantly impact the sperm's ability to reach and penetrate the egg during fertilization [32].

Sperm morphology: Oxidative stress can also contribute to abnormal sperm morphology, a condition called teratospermia. ROS can cause structural abnormalities in sperm, affecting their shape and impairing fertilization potential. Abnormal sperm morphology can hinder successful fertilization and decrease the chances of a healthy pregnancy [33].

Oxidative Stress and DNA Damage in Sperm

Sperm DNA is highly susceptible to oxidative stress, making it particularly vulnerable to DNA damage. Unlike other cells in the body, sperm cells have limited repair mechanisms, which further contributes to their susceptibility. The consequences of oxidative stress on sperm DNA can significantly affect fertility and reproductive outcomes. One of the major effects of oxidative stress on sperm DNA is DNA fragmentation. Oxidative stress can induce DNA strand breaks and fragmentation in sperm cells. This damage to the DNA structure can impair the ability of sperm to fertilize an egg successfully. Additionally, DNA fragmentation in sperm has been associated with an increased risk of embryo abnormalities, such as chromosomal rearrangements or genetic mutations. These abnormalities can affect embryo development, potentially leading to implantation failure or early pregnancy loss. Furthermore, DNA fragmentation has been shown to reduce pregnancy rates in assisted reproductive techniques [34].

In addition to DNA fragmentation, oxidative stress can also impact the overall integrity of sperm DNA. The damaging effects of oxidative stress can result in genetic mutations and chromosomal abnormalities within the sperm's DNA. These alterations can have severe consequences for embryonic development, potentially leading to developmental defects in offspring. Furthermore, the presence of genetic mutations or chromosomal abnormalities in sperm DNA can increase the risk of miscarriage. Studies have demonstrated a correlation between elevated oxidative stress levels and an increased likelihood of miscarriage due to sperm DNA damage [35].

Association Between Oxidative Stress and Male Infertility

There is growing evidence supporting the association between oxidative stress and male infertility. Studies have shown that infertile men often exhibit higher oxidative stress markers and lower antioxidant capacity than their fertile counterparts. Furthermore, conditions associated with male infertility, such as varicocele, infection, and exposure to environmental toxins, can increase oxidative stress levels in the male reproductive system [36]. Oxidative stress-induced damage to sperm, including reduced sperm count, impaired motility, abnormal morphology, and DNA fragmentation, can significantly impact male fertility. These effects can lead to difficulties in achieving pregnancy and an increased risk of recurrent miscarriages [37]. Understanding the link between oxidative stress and male infertility is essential for developing strategies to mitigate its effects. Antioxidant supplementation has emerged as a potential intervention to counteract oxidative stress and improve male reproductive health. In the next section, we will delve into the role of antioxidant supplementation and its effects on male fertility [38].

Antioxidant supplementation for male reproductive health

Overview of Antioxidant Supplementation and Effects on Male Fertility

Antioxidant supplementation involves the use of exogenous antioxidants, either as single antioxidants or in combinations, to enhance the body's antioxidant defense system and mitigate the effects of oxidative stress. It aims to restore the balance between ROS production and antioxidant protection, thus improving male reproductive health [39].

Improvement in sperm parameters: Several studies have demonstrated that antioxidant supplementation can improve sperm count, motility, and morphology. These improvements are attributed to reducing oxidative stress-induced damage to sperm cells [40-43].

Antioxidant supplementation has been proven to have a beneficial effect on reducing DNA fragmentation in sperm, which refers to the breaking or damaging of DNA strands within sperm cells. Additionally, studies have suggested that the pH of the sperm's solution could also play a role in this process. Maintaining an optimal pH level may contribute to sperm health and DNA integrity. As such, a comprehensive understanding of both antioxidant supplementation and the pH factor could hold promising implications for improving male fertility and reproductive outcomes. DNA fragmentation can lead to genetic abnormalities and impair the sperm's ability to fertilize an egg successfully. Antioxidants work by neutralizing harmful free radicals and reducing oxidative stress, a major cause of DNA damage in sperm. By protecting sperm DNA from oxidative damage, antioxidants help maintain DNA integrity, reducing DNA fragmentation [44].

In addition to reducing DNA fragmentation, antioxidant supplementation has also been associated with increased pregnancy rates in couples trying to conceive. Several studies have reported improved fertility outcomes, including higher pregnancy and live birth rates when the male partner received antioxidant supplements. This suggests that antioxidants play a crucial role in improving male fertility. By improving sperm quality and reducing oxidative stress, antioxidants create a more favorable environment for fertilization and embryo development, ultimately increasing the chances of successful pregnancy [45].

Types of Antioxidants Commonly Used in Supplementation

Vitamin C: Ascorbic acid, commonly known as vitamin C, is a potent antioxidant that can help reduce oxidative stress. Studies have shown that vitamin C supplementation can improve sperm parameters, including sperm count, motility, and morphology. It also can potentially reduce DNA damage in sperm cells, contributing to better reproductive outcomes [46].

Vitamin E: Alpha-tocopherol, a form of vitamin E, is another antioxidant extensively studied for its impact on male reproductive health. Vitamin E supplementation has improved sperm motility and reduced DNA damage in sperm cells. It helps protect sperm cell membranes from oxidative damage, maintaining their integrity and enhancing their ability to fertilize an egg [47].

Selenium is an essential trace mineral critical in the antioxidant defense system. It acts as a cofactor for various antioxidant enzymes, such as GPx, which help neutralize free radicals. Selenium supplementation has been linked to improved sperm function, including increased sperm motility and viability. It also contributes to the protection of sperm DNA integrity [48].

Coenzyme Q10: Coenzyme Q10, also known as ubiquinone, is a compound involved in cellular energy production. It also possesses antioxidant properties and helps protect cells from oxidative damage. Coenzyme Q10 supplementation has shown promise in improving sperm parameters, including sperm count, motility, and morphology. It may also reduce oxidative stress and improve sperm function [49].

Dosage and Duration Considerations for Antioxidant Supplementation

Consultation with healthcare professionals: It is important to consult with a healthcare professional or reproductive specialist before starting any antioxidant supplementation for male reproductive health. They can assess individual needs, consider underlying health conditions or medications, and provide personalized recommendations tailored to specific circumstances [41].

Dosage: The appropriate dosage of antioxidants varies depending on the specific antioxidant and formulation used. Following the recommended dosage guidelines provided on supplement labels or as advised by healthcare professionals is essential. Dosages can vary based on age, overall health, and specific fertility concerns [50].

Duration: Antioxidant supplementation often requires a certain duration to observe noticeable effects on sperm parameters. It is important to understand that improvements in sperm quality and fertility may take time. Consistency in taking the supplements as recommended and adhering to the prescribed duration is crucial for optimal outcomes. It is advisable not to expect immediate results and to give the supplements sufficient time to exert their effects [51].

Combination supplements: Some antioxidant supplements for male reproductive health may contain a combination of different antioxidants. These combinations are designed to leverage the synergistic effects of multiple antioxidants. When using combination supplements, it is important to follow the specific dosage and duration guidelines provided by the manufacturer or healthcare professional. Each antioxidant within the combination may have specific roles and interactions, and adherence to recommended guidelines ensures the intended benefits are realized [24]. It is important to note that while antioxidant supplementation holds promise, it may not be effective in all male infertility cases. The underlying causes of infertility should be carefully assessed, and supplementation should be considered as part of a comprehensive treatment approach.

Potential benefits and limitations of antioxidant supplementation

Potential Benefits of Antioxidant Supplementation on Sperm Quality and Fertility

Reduction of oxidative stress: Oxidative stress refers to an imbalance between the production of ROS and the body's ability to neutralize them with antioxidants. High levels of oxidative stress can negatively impact the male reproductive system, leading to sperm damage and impaired function. Antioxidants help combat oxidative stress by scavenging ROS and preventing their harmful effects on sperm cells. By reducing oxidative stress, antioxidants protect sperm cells from damage and promote optimal reproductive function [52].

Improved sperm parameters: Research studies have demonstrated that antioxidant supplementation can positively impact sperm parameters. Antioxidants help improve sperm count by preventing oxidative damage to sperm cells and reducing cell death (apoptosis). They also enhance sperm motility by preserving the structural integrity of sperm tails, allowing them to swim more efficiently. Additionally, antioxidants can improve sperm morphology by preventing structural abnormalities and promoting normal sperm development. Furthermore, antioxidants play a role in preserving DNA integrity in sperm cells, reducing the risk of DNA fragmentation and genetic abnormalities [53].

Enhanced fertility outcomes: The improvements in sperm parameters resulting from antioxidant supplementation can significantly affect fertility outcomes. By enhancing sperm quality, antioxidant supplementation can increase the chances of successful fertilization and embryo development. This, in turn, can improve pregnancy rates and overall reproductive success. Couples trying to conceive may benefit from incorporating antioxidant supplementation as part of their fertility treatment plan, especially if oxidative stress contributes to infertility [54].

Safety and ease of use: Antioxidant supplements are generally considered safe when used according to recommended dosages. Many antioxidant-rich foods and supplements are readily available, making them easily accessible for individuals seeking to improve male reproductive health. However, it is important to note that individual responses to antioxidant supplementation may vary, and it is always advisable to consult with a healthcare professional before starting any new supplementation regimen, particularly for individuals with underlying health conditions or taking medications [55].

Conflicting Research Findings and Limitations of Studies

Study design and methodology: The varying study designs, sample sizes, durations of supplementation, and assessment methods used in research studies on antioxidant supplementation can contribute to inconsistencies in findings. Differences in these factors make it challenging to draw definitive conclusions and generalize the results across different studies. Well-designed randomized controlled trials with larger sample sizes and standardized methodologies are needed to establish more robust evidence [56].

Heterogeneity of infertility causes: Male infertility is a complex condition with multiple underlying causes, including genetic factors, hormonal imbalances, structural abnormalities, and lifestyle factors. The effectiveness of antioxidant supplementation may vary depending on the cause of infertility. Studies often include participants with different etiologies of infertility, making it difficult to determine the precise impact of antioxidants in specific subgroups. Future research should aim to investigate the effects of antioxidants in well-defined subgroups based on the etiology of infertility [57].

Individual variations: Each individual has unique genetic makeup, lifestyle choices, environmental exposures, and overall health status, which can influence their response to antioxidant supplementation. Some individuals may have a greater susceptibility to oxidative stress or a higher baseline antioxidant status, which can affect their response to supplementation. Personalized approaches that consider individual variations may be necessary to optimize the benefits of antioxidant interventions [58].

Interaction with other treatments: Antioxidant supplementation is often used with other treatments for male infertility, such as lifestyle modifications, hormonal therapies, or assisted reproductive techniques. The interaction between antioxidant supplementation and these interventions may influence the observed outcomes in research studies. It is important to consider the potential synergistic or additive effects of antioxidant supplementation when evaluating the overall efficacy of combined treatment approaches [59].

Considering these limitations, further research is needed to address these challenges and provide more conclusive evidence regarding the benefits of antioxidant supplementation for male reproductive health. Well-designed studies with standardized protocols, larger sample sizes, and consideration of individual variations and underlying causes of infertility will contribute to a better understanding of the role of antioxidants in improving male reproductive function.

Considerations for Individual Variations and Underlying Causes of Infertility

Consultation with healthcare professionals: Before starting antioxidant supplementation for male reproductive health, it is crucial to consult with a healthcare professional or reproductive specialist. They can conduct a thorough evaluation, assess the causes of infertility, and determine if antioxidant supplementation is appropriate for the individual's unique situation [60].

Tailored treatment approach: Antioxidant supplementation should be considered part of a comprehensive treatment approach for male infertility. It is important to address any underlying causes of infertility, such as hormonal imbalances, structural abnormalities, or lifestyle factors. Combining antioxidant supplementation with other targeted interventions, such as lifestyle modifications and specific treatments, may improve reproductive outcomes overall [61].

Personalized dosage and duration: The optimal antioxidant supplementation dosage and duration may vary among individuals. Factors such as the severity of oxidative stress, baseline antioxidant status, and underlying health conditions can influence specific requirements. Healthcare professionals can provide personalized recommendations based on individual assessments, ensuring the dosage and duration of supplementation are tailored to the individual's needs [62].

Lifestyle modifications: Alongside antioxidant supplementation, a healthy lifestyle is crucial in optimizing male reproductive health. Regular physical exercise, a balanced and nutritious diet, stress reduction techniques, and avoidance of environmental toxins can help reduce oxidative stress and support overall reproductive function. These lifestyle modifications can complement the effects of antioxidant supplementation and contribute to improved outcomes [63].

Recommendations for antioxidant supplementation

Consulting a Healthcare Professional Before Starting Supplementation

Before initiating any antioxidant supplementation regimen for male reproductive health, consulting with a healthcare professional or reproductive specialist is strongly recommended. They can assess individual needs, evaluate underlying causes of infertility, and provide personalized recommendations. Healthcare professionals can guide patients on the appropriate dosage, duration, and potential benefits or risks associated with antioxidant supplementation [55].

Potential Risks and Interactions of Antioxidant Supplements

Overdose or toxicity: While antioxidants are generally safe, excessive amounts of certain antioxidants can lead to toxicity and adverse effects. For example, high doses of vitamin E or selenium can be harmful. It is important to adhere to recommended dosages and avoid exceeding the recommended limits without medical supervision [64].

Interaction with medications: Antioxidant supplements can interact with certain medications, including blood thinners, chemotherapy drugs, and others. These interactions can affect the efficacy or safety of antioxidant supplements and medications. It is crucial to inform healthcare professionals about all medications being taken, including over-the-counter supplements, to minimize the risk of potential interactions [65].

Individual allergies or sensitivities: Some individuals may have allergies or sensitivities to specific antioxidants or ingredients used in antioxidant supplements. It is important to read labels carefully, checking for potential allergens or known sensitivities. If there is uncertainty or a history of allergies, it is advisable to consult a healthcare professional before starting any antioxidant supplementation [66].

Guidelines for Choosing and Using Antioxidant Supplements Effectively

Quality and reputation: Choosing products from reputable manufacturers who adhere to good manufacturing practices (GMPs) is important when selecting antioxidant supplements. Look for supplements that undergo third-party testing for quality, purity, and potency [67].

Comprehensive formulations: Consider antioxidant supplements that provide a comprehensive blend of antioxidants. A diverse combination of antioxidants can target different sources of oxidative stress and offer synergistic effects. Look for supplements that include a range of antioxidants such as vitamins C and E, selenium, and other phytonutrients [68].

Bioavailability and absorption: Opt for supplements that utilize forms of antioxidants with high bioavailability and absorption rates. Other substances, such as healthy fats or certain food combinations, may better absorb some antioxidants. Look for supplements incorporating bio-enhancers or advanced delivery systems for improved absorption [69].

Timing and consistency: Follow the recommended dosage instructions provided by the manufacturer or healthcare professional. Consistency is important in reaping the potential benefits of antioxidant supplementation. Establish a regular supplementation schedule and adhere to it consistently [70].

Monitoring and evaluation: Regular monitoring of sperm parameters under the guidance of a healthcare professional, can help assess the effectiveness of antioxidant supplementation. Periodic evaluation of sperm count, motility, morphology, and DNA fragmentation can provide valuable insights into the impact of antioxidants on reproductive health. Based on individual responses and progress, adjustments to the supplementation regimen may be necessary [71].

Lifestyle modifications: Antioxidant supplementation should be complemented by a healthy lifestyle. Focus on adopting a balanced diet rich in fruits, vegetables, whole grains, and lean proteins. Engage in regular physical activity, manage stress effectively, and avoid exposure to environmental toxins. These lifestyle modifications can synergize with antioxidant supplementation and support reproductive health [72].

Remember that antioxidant supplementation should be viewed as part of a comprehensive approach to male reproductive health rather than a standalone solution. It is essential to address the underlying causes of infertility and consider other treatments or interventions recommended by healthcare professionals. By following these recommendations, individuals can make informed decisions regarding antioxidant supplementation and optimize their chances of improving male reproductive function and fertility outcomes.

Lifestyle factors to reduce oxidative stress

Importance of a Healthy Lifestyle in Reducing Oxidative Stress

Regular exercise: Regular physical activity and exercise have numerous benefits for reducing oxidative stress. Moderate-intensity aerobic exercises, such as brisk walking, jogging, cycling, or swimming, have been shown to enhance the body's antioxidant defense systems. Exercise stimulates the production of antioxidant enzymes, improves blood flow, and enhances the removal of harmful byproducts associated with oxidative stress. Incorporating regular exercise into your routine can help mitigate oxidative damage and promote overall health [73].

Stress management: Chronic stress can increase oxidative stress levels in the body. When we experience stress, the body produces stress hormones that can generate ROS and disrupt the balance of antioxidants. Implementing stress management techniques can help reduce stress levels and minimize oxidative stress. Practices such as mindfulness meditation, deep breathing exercises, yoga, or engaging in hobbies and activities that promote relaxation can effectively lower stress levels and support overall well-being [74].

Adequate sleep: Quality sleep is crucial in maintaining optimal health, including managing oxidative stress. During sleep, the body undergoes important restorative processes that help combat oxidative damage and restore antioxidant balance. Sleep deprivation or disturbances can disrupt these processes, increasing oxidative stress. Aim for seven to nine hours of uninterrupted sleep per night to support the body's natural antioxidant defense mechanisms. Establishing a consistent sleep routine, creating a conducive sleep environment, and practicing good sleep hygiene can contribute to better sleep quality and an overall reduction in oxidative stress levels [75].

Dietary and Nutritional Recommendations for Reducing Oxidative Stress

Antioxidant-rich foods: To promote a healthy diet, it is important to incorporate a wide range of antioxidant-rich foods. These include colorful fruits and vegetables like berries, citrus fruits, leafy greens, and cruciferous vegetables. Such foods contain various antioxidants, such as vitamins C and E, beta-carotene, and phytochemicals. These antioxidants play a crucial role in combating oxidative stress, which can lead to cellular damage and various health issues [76].

Omega-3 fatty acids: Another important dietary component to consider is omega-3 fatty acids. These can be found in fatty fish (salmon, mackerel, sardines), flaxseeds, chia seeds, and walnuts. Omega-3 fatty acids are known for their anti-inflammatory properties and ability to reduce oxidative stress. Incorporating these foods into your diet can promote a healthier balance within your body [77].

Mediterranean diet: The Mediterranean diet is a dietary pattern that emphasizes whole grains, lean proteins, healthy fats like olive oil and nuts, and various fruits and vegetables. This diet has been associated with reduced levels of oxidative stress and improved overall health. By following this eating pattern, you can enjoy a wide range of antioxidant-rich foods and benefit from the anti-inflammatory effects of healthy fats [78].

Limiting processed foods and trans fats: It is essential to reduce the intake of processed foods and those high in trans fats. These foods have been linked to increased inflammation and oxidative stress in the body. Opting for whole, unprocessed foods whenever possible can help minimize these harmful effects and provide your body with the necessary nutrients [79].

Hydration: Adequate hydration is important for overall health, including reducing oxidative stress. By consuming adequate water and minimizing the intake of sugary beverages, you can support your body's natural detoxification processes and help maintain cellular health. Staying hydrated allows your body to function optimally and helps flush out toxins [80].

Other Lifestyle Modifications for Optimizing Male Reproductive Health

Avoiding tobacco and alcohol: Smoking tobacco and excessive alcohol consumption have been linked to increased oxidative stress and detrimental effects on sperm quality. Tobacco smoke contains harmful chemicals that generate free radicals, leading to oxidative damage. Excessive alcohol consumption can disrupt the body's antioxidant balance and increase the production of free radicals. Quitting smoking and limiting alcohol intake can positively impact reproductive health and reduce oxidative stress levels [81].

Environmental toxins: Reduce exposure to environmental toxins that can contribute to oxidative stress. Pollutants, pesticides, and industrial chemicals in the air, water, and food sources can generate free radicals and damage sperm cells. Minimize exposure to these toxins by avoiding areas with heavy pollution, consuming organic produce, and using natural cleaning and personal care products. Taking necessary precautions in occupational settings where exposure to chemicals is common is also important [82].

Limiting heat exposure: Prolonged exposure to excessive heat can harm sperm quality. High temperatures, such as those experienced in saunas or hot tubs or by wearing tight underwear, can impair sperm production and motility. Opt for loose-fitting underwear and avoid prolonged exposure to hot environments to maintain optimal reproductive function [83].

Weight management: Maintaining healthy body weight is important for overall health and reproductive function. Obesity has been associated with increased oxidative stress, hormonal imbalances, and inflammation, which can negatively impact fertility. Strive for a healthy weight through a balanced diet, nutrient-rich foods, and regular physical activity. Weight loss in overweight or obese individuals can help reduce oxidative stress and improve reproductive health [84]. By incorporating these lifestyle modifications, individuals can reduce oxidative stress levels, promote well-being, and optimize male reproductive health. These lifestyle factors and appropriate antioxidant supplementation can synergistically support fertility and reproductive function.

## Conclusions

In conclusion, oxidative stress profoundly impacts male reproductive function, and its detrimental effects on sperm quality and fertility are well-documented. Oxidative stress disrupts the delicate balance between ROS production and antioxidant defense mechanisms, leading to oxidative damage to sperm cells and impairing their viability and function. Antioxidant supplementation has emerged as a potential therapeutic approach to mitigate the harmful effects of oxidative stress on male reproductive health. By providing exogenous antioxidants, it is possible to scavenge excess ROS, restore the redox balance, and protect sperm cells from oxidative damage. Numerous studies have reported positive outcomes with antioxidant supplementation, demonstrating improvements in sperm count, motility, morphology, DNA integrity, and fertility outcomes. However, conflicting research findings and limitations exist within the field, highlighting the need for further investigation. Variations in study design, sample sizes, duration of supplementation, and assessment methods contribute to the inconsistent outcomes observed in research studies. Additionally, the heterogeneity of infertility causes and individual variations in response to supplementation further complicate the interpretation of results. It is important to emphasize that antioxidant supplementation should be considered part of a comprehensive approach to male reproductive health. Consulting with healthcare professionals or reproductive specialists is crucial to identify the underlying causes of infertility and determining the appropriateness of antioxidant supplementation. Additionally, individuals should strive to adopt a healthy lifestyle, including regular exercise, a balanced diet rich in antioxidant-rich foods, stress management, and avoidance of environmental toxins.
